# Editorial: Biotechnology for Arsenic Detection and Bioremediation

**DOI:** 10.3389/fmicb.2021.743109

**Published:** 2021-10-05

**Authors:** Natalia González-Benítez, Gonzalo Durante-Rodríguez, Manoj Kumar, Manuel Carmona

**Affiliations:** ^1^Department of Biology, Geology, Physics and Inorganic Chemistry, Universidad Rey Juan Carlos, Móstoles, Spain; ^2^Microbial and Plant Microbiology Department, Centro de Investigaciones Biológicas Margarita Salas-CSIC, Madrid, Spain; ^3^Environmental Toxicology Group, CSIR-Indian Institute of Toxicology Research, Lucknow, India

**Keywords:** arsenic, arsenic resistance, phytoremediation, endophyte, arsenic bioremediation

Arsenic contamination by natural causes or industrial activities is a major environmental concern, and treatment of contaminated environments is needed to protect ecosystems and minimize the risk for human health (Mead, 2005). Industrial activities have increased the environmental concentration of arsenic, leading to biodiversity deterioration. Several physicochemical techniques exist for the clean-up of arsenic-contaminated systems, however most of them are expensive and not eco-friendly. In the light of the UN's Sustainable Development Goals, bioremediation has been highlighted as a key technology to achieve these goals (Upadhyay et al., 2018). Arsenic and microorganisms are coexisting on Earth from many million years, and as result, bacteria displayed wide distribution and extreme diversity of arsenic mechanisms of resistance to arsenic (Andres and Bertin, 2016). Understanding of the molecular determinants underlying arsenic resistance is increasing continuously and that knowledge can be used to develop improved strategic frameworks for biotechnology-based bioremediation. Also, understanding the role of microorganisms in the arsenic removal in plants under arsenic stress, will allow for the development of novel arsenic bioremediation strategies, both *in situ* and *ex situ*. This Research Topic is devoted to the description of current advance in microbial biotechnology for arsenic elimination. It includes the depiction of molecular determinants responsible for the arsenic resistance, new isolation of microorganisms harboring molecular determinants useful for arsenic elimination, and the association between arsenic resistant bacteria with plants that can be used in phytoremediation strategies. Also the impact on the microbiota caused by several bioremediation strategies employed in arsenic removal were analyzed (Figure 1).

**Figure 1:**
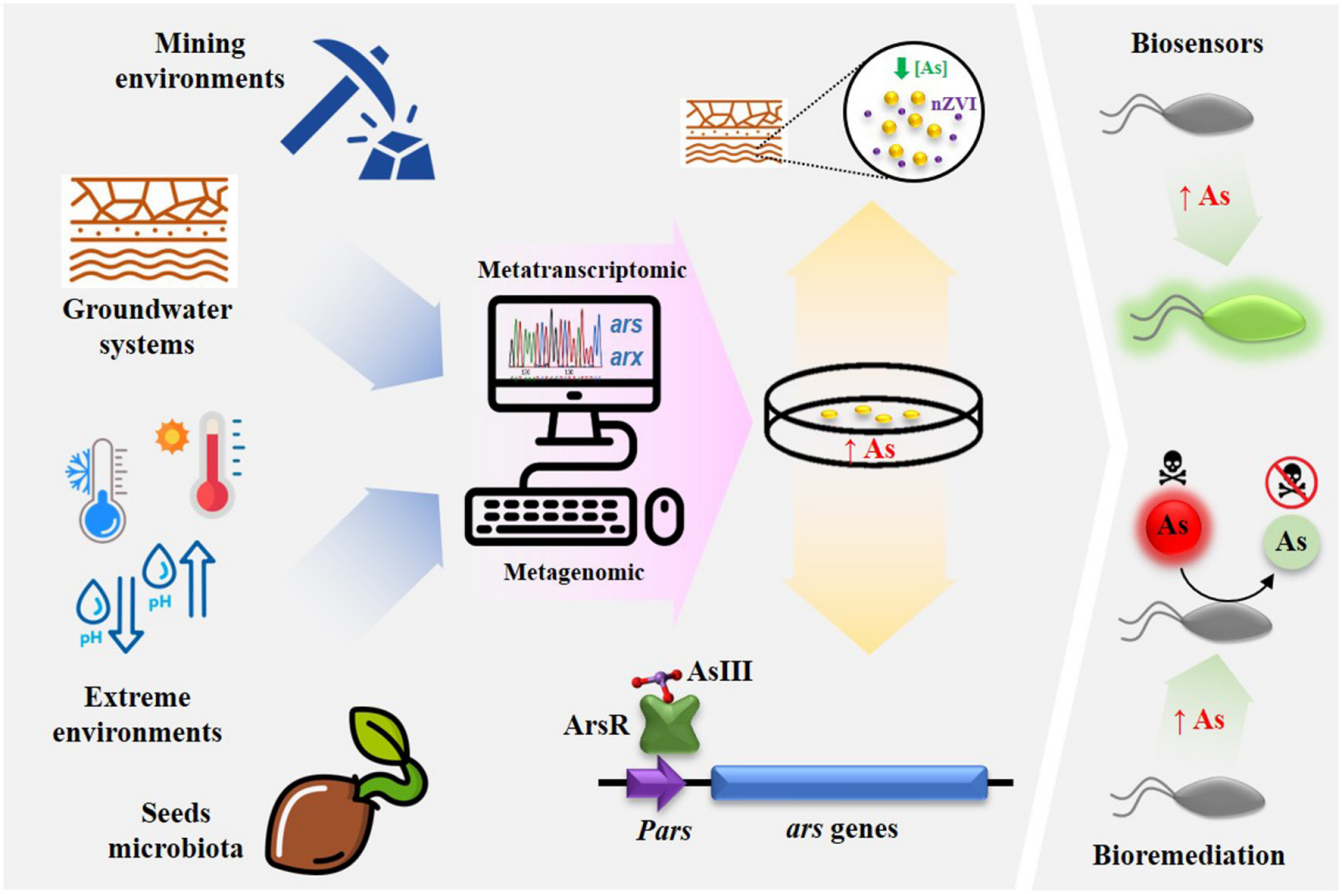
Conceptual summary depicting different environments to detect microorganisms harboring As tolerance strategies are important tools for arsenic detection (Biosensensors) and bioremediation.

Arsenic mobilization in groundwater systems and extreme environments is driven by a variety of functionally diverse microorganisms and complex interconnections between different physicochemical factors. In order to unravel this great ecosystem complexity, Zecchin et al. analyzed groundwaters with varying background concentrations and speciation of arsenic in one of the most populated areas in Europe affected by metalloid contamination. High-throughput Illumina 16S rRNA gene sequencing, CARD-FISH and enrichment of arsenic-transforming consortia showed that among the analyzed groundwaters, diverse microbial communities were present, both in terms of diversity and functionality. Several uncharacterized bacteria and As(III)-oxidizing consortia were identified. The results presented suggest that contaminated aquifers host unexplored microbial populations that provide essential ecosystem services and microbial with biotechnology purpose. Furthermore, in order to find and characterize microorganisms in extreme environments, Aulitto et al. focused its study on the isolation and genomic exploration of a new arsenic-tolerant microorganism, classified as *Alicyclobacillus mali* FL18, isolated in an arsenic-rich hot spring near the Campi Flegrei volcano (Italy). *A. mali* FL18 showed a good tolerance to arsenite (MIC value of 41 mM), as well as to other metals. Moreover, the strain showed a high resistance to bacitracin and ciprofloxacin, suggesting that the extreme environment has positively selected multiple resistances to different toxic compounds. This work provides insights into the heavy metal tolerance and antibiotic susceptibility of an *Alicyclobacillus* strain and highlights its putative molecular determinants.

The importance of monitoring the impact of bioremediation strategies on ecosystems is the leitmotiv of the following publication. The work of Castaño et al. analyses the impact of Nanoscale Zero-Valent Iron (nZVI) on microbiota involved in arsenic elimination from environments. nZVI is a nanomaterial widely used to remove a broad range of metal(loid)s and organic contaminants from soil and groundwater. In some cases, this material alters the taxonomic and functional composition of the bacterial communities present in these matrices; however, conclusive data were not presented prior to this publication. Microbiological (cultured and total bacterial diversity) parameters were monitored before and after nZVI application over 6 months in an abandoned fertilizer factory, mainly polluted with arsenic. A substantial decrease in the concentration of arsenic during the first days of treatment was observed, although strong fluctuations were subsequently detected in most of the wells throughout the 6-month experiment. A more complete study of the structure and diversity of the bacterial community in the groundwater confirmed significant alterations in its composition, with a reduction in richness and diversity after treatment with nZVI.

In mining environments, treatment peatlands are used to remove arsenic and other contaminants. However, the microbiota and the arsenic turnover process within these environments are poorly studied. Ziegelhöfer and Kujala have showed the taxonomic and functional diversity of psychrotolerant arsenic microorganisms from a subarctic peatland affected by mining environment in Finnish Lapland. To achieve this purpose, metagenomics, metatranscriptomics, and culture-dependent approaches such as dilution-to-extinction techniques have been used. These environments have showed that, most of operational taxonomic units (OTUs) were not abundant (rare biosphere) and arsenic tolerant (arsenite-oxidizing and arsenate-respiring). Some of the OTUs identified belonged to genera *Pseudomonas, Polaromonas, Aeromonas, Brevundimonas, Ancylobacter*, and *Rhodoferax* which respire arsenate and will serve as model organisms for further studies of arsenic-metabolizing microbial activities in peatlands. Concluding, authors have proposed that these environments and mainly the rare biosphere might contribute to arsenic turnover playing an important role in the treatment of arsenic-contaminated environments. The following study also considered the rare biosphere as an important source of microbial diversity and functionality. González-Benítez et al. study has described the role of horizontal and vertical transmission of bacterial microbiota and how the stressors such as arsenic drive seeds microbiota from an arsenic-tolerant plant such as *Jasione montana*. Culture-independent techniques (High-throughput Illumina sequencing) showed *Jasione* bacterial endophytes core composed by *Pseudomonas, Ralstonia, Undibacterium, Cutibacterium, Kocuria*, and family Comamonadaceae. Most of them were previously identified as plant promoting growth bacteria (PPGB) or tolerant to arsenic. The OTUs *Rhodococcus* spp. were identified as exclusive endophyte associated to *J. montana*. The authors isolated *Rhodococcus rhodochrous* from *J. montana* collected from highly arsenic contaminated soils. This strain was identified as PPGB and arsenic-resistant and used in bioaugmentation approach. From a functional point of view, *R. rhodochrous* showed to colonize efficiently both *Jasione* species, modifying the response to arsenic stress improving the arsenic tolerance of *J. sessiliflora*. Despite most of the OTUs identified inside *Jasione* seeds belonged to rare microbiota, they represent a large bacterial reservoir offering important physiological and ecological traits to the plant host. Moreover, plant seed endomicrobiota should be considered a resource for exploitation given its potential applications and utility to environmental remediation, sustainable agriculture, and biotechnology.

The previous studies tackle more prospective projects that are very well-complemented by more detailed and molecular approaches. Thereby, Durante-Rodríguez et al. study the transcriptional regulation of the apparently unnecessary duplication of the two *ars* clusters borne by the soil bacterium *Pseudomonas putida* KT2440. *In vitro* experiments revealed a cross talk between the two repressors ArsR1/ArsR2 and the respective promoters within a different set of parameters, although non-identical binding sequences were characterized. The authors propose that this regulatory frame fits as a particular type of bifan motif, whose distinct regulatory architecture could be the basis of an adaptive advantage that favors the maintenance of the two proteins as separate repressors.

With this Research Topic, we described new microorganisms, and plant-bacteria association characterized by their ability to survive in arsenic polluted environments. Although the described microorganisms are very interesting, further in deep research are needed to implement their employment in arsenic removal technologies. Also, the understanding of the molecular determinant responsible for arsenic resistance might be important in using synthetic biology technologies to construct better chassis for bioremediation or biosensors to detect contamination in the ecosystems. Finally, the inclusion of technologies to monitor the arsenic removal process and their impact in the ecosystems will implement a productive future for arsenic bioremediation.

## Author Contributions

All authors of this article performed equal contributions to the work and approved it for publication.

## Funding

This work was supported by grants PID2019-110612RB-I00 from Ministry of Science and Innovation of Spain and DRIADES Project from Universidad Rey Juan Carlos (AYUDA PUENTE).

## Conflict of Interest

The authors declare that the research was conducted in the absence of any commercial or financial relationships that could be construed as a potential conflict of interest.

## Publisher's Note

All claims expressed in this article are solely those of the authors and do not necessarily represent those of their affiliated organizations, or those of the publisher, the editors and the reviewers. Any product that may be evaluated in this article, or claim that may be made by its manufacturer, is not guaranteed or endorsed by the publisher.
